# The Comparison of Dietary Behaviors among Rural Controlled and Uncontrolled Hypertensive Patients

**DOI:** 10.1155/2016/7086418

**Published:** 2016-07-19

**Authors:** Aziz Kamran, Ali Akbar Shekarchi, Elham Sharifian, Heshmatolah Heydari

**Affiliations:** ^1^Public Health Department, School of Health, Ardabil University of Medical Sciences, Ardabil 5614995935, Iran; ^2^Public Health Department, Khalkhal Faculty of Medical Sciences, Ardabil University of Medical Sciences, Ardabil 5614995935, Iran; ^3^Nursing Department, Khalkhal Faculty of Medical Sciences, Ardabil University of Medical Sciences, Ardabil 5614995935, Iran; ^4^Community Health Nursing Department, School of Nursing and Midwifery, Lorestan University of Medical Sciences, Khorramabad 381351698, Iran; ^5^Community Health Nursing Department, School of Nursing and Midwifery, Tehran University of Medical Sciences, Tehran 1419733171, Iran

## Abstract

Nutrition is a dominant peripheral factor in increasing blood pressure; however, little information is available about the nutritional status of hypertensive patients in Iran. This study aimed to compare nutritional behaviors of the rural controlled and uncontrolled hypertensive patients and to determine the predictive power of nutritional behaviors from blood pressure. This cross-sectional study was conducted on 671 rural hypertensive patients, using multistage random sampling method in Ardabil city in 2013. Data were collected by a 3-day food record questionnaire. Nutritional data were extracted by Nutritionist 4 software and analyzed by the SPSS 18 software using Pearson correlation, multiple linear regression, ANOVA, and independent *t*-test. A significant difference was observed in the means of fat intake, cholesterol, saturated fat, sodium, energy, calcium, vitamin C, fiber, and nutritional knowledge between controlled and uncontrolled groups. In the controlled group, sodium, saturated fats, vitamin C, calcium, and energy intake explained 30.6% of the variations in blood pressure and, in the uncontrolled group, sodium, carbohydrate, fiber intake, and nutritional knowledge explained 83% of the variations in blood pressure. There was a significant difference in the nutritional behavior between the two groups and changes in blood pressure could be explained significantly by nutritional behaviors.

## 1. Introduction

Current recommendations for the management and prevention of hypertension are based on the lifestyle changes along with medication [1] and, among these lifestyle changes, much attention has been paid to nutrition. Actually, nutrition is a dominant peripheral factor in increasing blood pressure [2]. Population based studies have shown that the consumption of fruits, vegetables, and dairy products has beneficial effects on blood pressure [3] and increased consumption of fruits and vegetables lowers blood pressure [3–5]. In addition, several studies have shown that adding low-fat dairy products in the nutrition pattern can cause a significant decrease in blood pressure [4, 6]. In other studies, a significant relationship has been observed between increased consumption of milk and reduced blood pressure and coronary artery disease [7]. Electrolytes also play an important role in regulating blood pressure and multiple lines of evidence suggest that systolic blood pressure is directly related to the high sodium protein and alcohol intake and is inversely associated with the consumption of potassium and calcium, and diastolic blood pressure is inversely associated with the consumption of potassium and calcium [8]. Majority of patients do not adhere to the daily recommended amount of calcium and sodium [9] and lack of self-care in hypertensive patients' adherence to nutritional advice has been reported in several studies [10–13]. Also, there is no complete information on the self-care nutritional status of the patients, and the available conducted studies on this issue are not desirable [14]; for instance, in the study conducted by Haghighatdoost et al. [15] and Khaledifar et al. [16], the sodium intake was high. The authors could not find many studies about the nutritional status of rural patients, especially on the amount of salt, total fat, saturated fat, and other macronutrients' consumption, and, in the only found study, the average sodium intake of rural patients was reported as 2,989 milligrams per day [17] which is much more than the recommended dose for these patients. To consider the crucial role of nutrition in the control of hypertension, research about dietary patterns of rural patients is necessary. Therefore, the present study is designed to provide complete information on the consumption status of macro- and micronutrients, compares the dietary patterns of patients with controlled and uncontrolled hypertension, and, consequently, provides useful information for policymakers and healthcare professionals.

## 2. Methods

This is a cross-sectional study which was conducted in 2014 on the population of patients with hypertension who were under the coverage of rural health centers of Ardabil city. The study sample consisted of controlled and uncontrolled patients who were selected through a multistage sampling method and in total 700 patients who had hypertension history were selected; then, patients who had systolic blood pressure of 140 mmg and higher were merged in the uncontrolled group and patients who had systolic blood pressure of 139 mmg and lower were merged in the controlled group.

Thus, at the first stage, among 13 rural health centers, six centers were selected randomly, and then 700 patients covered by the selected centers were eligible for study. Inclusion criteria included diagnosed hypertension, having a health file at the health center, lack of severe complications of disease, 30–65-year-old patients, literacy, and willingness to participate in the study. Exclusion criteria included unwillingness to continue participating in the research. When the patients were selected, the aim and the methodology of the study were explained to them and informed consent was obtained.

### 2.1. Study Tool

A questionnaire was employed in this study. The questionnaire comprised (1) demographic information questionnaire which included gender, age, educational level, systolic and diastolic blood pressure, family history, and duration of illness; (2) standard nutrition awareness questionnaire (10 questions with true/false options) [18]; and (3) the questionnaires of 3-day food consumption food record. In the scoring of the knowledge questions, correct answers were represented by 2 points and incorrect answer was represented by zero points.

### 2.2. Data Collection

Data was collected by questionnaires in every health center by health workers that are informed about the aim and methodology of the study and they were responsible for gathering the data. The first and second parts of the questionnaire were completed in the health center, by structured face-to-face interviews with the participants. To complete the third part of the questionnaire, the 3-day food consumption record, cases were trained on recording foods consumption in the questionnaire for a week (two working days and a holiday) and then came back to complete the questionnaire in the health center. The list of consumed foods on three days of the week was recorded. After collecting the questionnaire, 29 patients, due to lack of accurate data in their questionnaires of 3-day food consumption record, were excluded and, finally, 671 cases were eligible for analysis.

### 2.3. Data Analysis

The dietary data were extracted through Nutritionist 4 (N4) software which included macronutrients (amount of fat, cholesterol, protein, carbohydrate, and total energy intake) and were analyzed through Excel 2007 software, SPSS 18 software, Pearson correlation, multiple linear regression, independent *t*-test, one-way ANOVA, and Chi-square by considering the significance level of 0.05.

## 3. Results

Among a total number of 671 eligible cases, 433 patients were in the controlled group and 238 were in the uncontrolled group. There was no significant difference in mean age and duration of disease in both groups (*P* > 0.05). Also, there was no significant difference in positive family history, financial income, and literacy level (*P* > 0.05). However, a significant difference was observed between the two groups in gender and BMI. The mean BMI in the uncontrolled group of patients was more than that in the controlled group (see Table 1).

The mean of the systolic blood pressure in the controlled group was 133.3 and in the uncontrolled group was 153.5 mmHg. Average consumption of fat, cholesterol, saturated fat, protein, carbohydrates, sodium, and energy intake in patients with uncontrolled disease were significantly higher and the intake of calcium, vitamin C, dietary fiber, and nutritional awareness were significantly higher in the hypertension- (HT-) controlled group (Table 2).

In the HT-controlled group, systolic blood pressure was significantly associated positively with fat, saturated fat, cholesterol, calories intake, and consumed sodium and was significantly inversely associated with vitamin C and dietary fiber consumption (Table 3).

In the uncontrolled group, systolic blood pressure had a significant direct relationship with sodium intake and no significant association was found with other micro- and macronutrients (Table 4).

In the HT-controlled group, sodium intake can be explained as or by 19.5% of the variance of systolic blood pressure but in the uncontrolled group this was explained by 71.7% and in total, in model 3 of multiple linear regression, sodium, saturated fats, vitamin C, calcium, and energy intake in HT-controlled group had 30.6% predictive power, and, in the uncontrolled group, sodium, carbohydrates, and consumed dietary fiber along with dietary knowledge can be explained by or as 83% of changes in systolic blood pressure (Table 5).

## 4. Discussion

Nutrition has an undeniable role in the control and prevention of hypertension but, in fact, adherence to dietary recommendations among these patients is low [19]. Epstein et al. [20], Racine et al. [21], and Troyer et al. [22] reported that the score of adherence to a healthy diet was low; also, in the study of Couch et al., only 21% of the individuals reached the proposed goals for the consumption of fruits and vegetables, fat, and saturated fat in the period of 3 months [23].

In this study, the mean fat intake in both groups of patients was more than the provided recommendations for these patients in a similar Iranian diet [24]. This amount of fat intake is more than the recommended amount of fat in the Dietary Approach to Stop Hypertension (DASH) [19]. Although fat intake in these patients is high, reducing the amount of fat intake in these patients is very important, because high fat diet increases the level of blood pressure [25]. Study findings revealed that fat intake had a significant positive correlation with systolic blood pressure of HT-controlled group which was in line with the study of Colín-Ramírez et al. [26]. The average of saturated fat intake in the uncontrolled group is more than the recommended amount in the DASH [19]. The American Heart Association recommends that less than 7% of calories should be from saturated fat [27]. Study findings also indicated that saturated fat intake was positively associated with systolic blood pressure in the HT-controlled group which is in line with similar studies which reported a significant positive relationship between saturated fat intake and blood pressure [28].

The average of carbohydrate intake in the uncontrolled group was significantly greater and, in general, the mean consumption of carbohydrate was consistent with a similar study conducted on patients of the same age and weight in which the amount of carbohydrates was 314 grams [29]. The findings showed that the carbohydrate intake was positively associated with systolic blood pressure in the HT-controlled group. Multiple lines of evidence also suggest that the size and type of carbohydrates consumed can affect the blood pressure [2]. In the other studies, which specifically investigated the relationship between carbohydrate intake and blood pressure, conflicting results have been obtained; in another study, there was a direct relationship [30] or sometimes inverse correlation and in other cases no significant correlation has been found [2], and in Morris's study, no reduction was observed in blood pressure through the increased carbohydrate and low-fat intake [31]. Average intake of protein in uncontrolled and HT-controlled groups was, respectively, 112 and 109 grams per day and this deferential was significant statistically. In Ijarotimi and Keshinro's study which was conducted on patients of the same age and weight, the amount of protein intake was 1.93 grams [29]. The type of consumed protein (animal, plant) may have different effects on blood pressure [32]. Also, He et al. examined the effects of soy and milk's proteins on blood pressure; they observed significant differences between effects these two proteins on hypertension [33] and, in another study, the replacement of carbohydrates with plant protein lowered blood pressure [34].

Average dietary fiber intake was significantly higher in the HT-controlled group; however, its amount was less than the recommended amount in the DASH (30 mg daily). In a similar study on patients who almost were of the same age and weight, the dietary fiber intake was 5.6 grams [29] which is consistent with the study funding of Boeckner et al. [35] where the average fiber intake and fruit and vegetable consumption of rural women were less than the recommended amount. In this study, the amount of dietary fiber intake had a significant inverse relationship with systolic blood pressure in the HT-controlled group and conducted studies show that the prevalence of hypertension in people with high fruit and vegetable intake was low and inversely associated with blood pressure [36, 37]. Also in the present study there was a significant inverse relationship between the average intake of vitamin C and systolic blood pressure in the HT-controlled group. Vitamin C is a powerful antioxidant and is known as a vasodilator [38]. Meta-analysis of 29 studies showed that vitamin C supplementation can reduce systolic blood pressure up to 5 mmHg [39]. Thus, the inverse relationship between vitamin C amount and cardiovascular complications may be related to lowering blood pressure [40]. Agriculture is the main occupation of the studied rural area and most of the foods with high content of vitamin C such as kale, fresh thyme, cauliflower, oranges, tomatoes, and kiwi are accessible and the authors recommend that patients with uncontrolled hypertension consume these types of food.

In this study, the mean energy intake was significantly higher in the uncontrolled group and this amount of energy intake due to the seasonal condition of the study is more than the required daily amount which is in line with the study of Boeckner et al. in which the mean energy intake of rural women was more than the recommended amount [35]. Black halwa, broth, haleem, and butter are some of the foods that uncontrolled hypertensive patients consumed significantly higher than HT-controlled group patients.

The average of sodium intake in the uncontrolled group was significantly higher and in both groups was more than its recommended amount. In Khosravi et al.'s study on the prehypertension patients, the salt intake was 12.5 grams per day which was twice the recommended amount [41]. Similar results were obtained in the study of Haghighatdoost et al. [15] and Kojuri and Rahimi [42]. Although limiting the intake of sodium is particularly important in patients with hypertension, reducing salt intake to 3-4 grams per day can greatly lower blood pressure and also is beneficial in reducing the risk of cardiovascular diseases [43]. In this study, sodium intake had a direct relationship with systolic blood pressure in both groups and this relationship was stronger in the uncontrolled group. Based on the lines of evidence, the relationship between sodium intake and blood pressure is linear and by reducing salt intake the risk of diseases decreases [44, 45]. Some of the common foods with high sodium in the studied area are Dovga, local cheese, and table salt, which is another problem for reducing sodium intake.

The mean calcium intake in both groups was less than the recommended amount which was in line with the study of Esmaillzadeh et al. in which hypertensive patients had less calcium intake than the recommended amount [46]. This is also in line with Boeckner et al.'s [35] study that indicated that the amount of nutrients intake is dependent on various factors, including socioeconomic factors, age, differences, and cultural and geographical factors [46]. Also, in this study, the amount of potassium consumption was less than the recommended amount which was in line with the study of Esmaillzadeh et al. in which patients with high blood pressure had less potassium intake than the recommended amount [46]. The fact is that potassium consumption in most countries is less than the recommended amount [47]. In communities that have higher potassium intake, however, the mean incidence of hypertension is low [46, 48]. The authors recommend high potassium content foods such as greens, cantaloupe, carrots, date, banana, and beets which are accessible easily in most of the rural area, to be consumed more. One of the limitations of this study is that it is conducted as cross-sectional which by itself makes the determination of causal relations difficult. In contrast, this study is the first comparative study of dietary patterns of uncontrolled and controlled hypertension patients in Iran, which provides valuable information for researchers and authorities, who are responsible for the planning of health services. Multiple regression analysis performed with a desired number of samples is a positive point of this study. In addition, using three-day food record decreased the underestimation possibility; on the other hand, estimates from the 3-day diet records better agreed with actual intake.

## 5. Conclusion and Recommendations

There was a significant difference in dietary behavior of both groups, and dietary behaviors had a strong role in the prediction of hypertensive changes of patients; therefore, more attention should be paid to the dietary behavior of patients and comprehensive and stable instructions are most important in designing the necessary interventions by planners and professionals of healthcare systems.

## Figures and Tables

**Table 1:** Demographic characteristics of patients participating in the study in two controlled and uncontrolled groups.

Variable	Controlled group	Uncontrolled group	*P* value
	Mean (SD)	Mean (SD)	
Age (years)	50.2 ± 6.6	50.4 ± 5.9	*P* = 0.6
Disease duration (years)	5.9 ± 4.0	5.5 ± 3.2	*P* = 0.2
BMI (kg/m^2^)	29.2 ± 4.1	30.1 ± 5.60	*P* = 0.04

	Number (%)	Number (%)	

Literacy^*∗∗*^			
Primary school	335 (77.4)	173 (73.1)	*P* = 0.324
Middle school	89 (20.6)	58 (23.5)	
High school	9 (2.1)	8 (3.4)	

Family history			
Yes	215 (49.7)	116 (48.7)	*P* = 0.8
No	218 (50.3)	122 (51.3)	

Sex			
Male	95 (21.9)	74 (31.1)	*P* = 0.001
Female	338 (78.1)	164 (68.9)	

^*∗∗*^Primary school starts from the first to the fifth grade. Middle school goes from the sixth to the eighth grade. High school is the last three years and is required to enter into higher education.

**Table 2:** Mean blood pressure and dietary factors in two groups of patients with hypertension.

	Uncontrolled group	Controlled group	*P* value
Systolic BP	153.5 ± 5.1	133.3 ± 7.5	*P* < 0.001
Fat	108.6 ± 19.2	83 ± 27.6	*P* < 0.001
Potassium	184.8 ± 21.4	174.7 ± 19.3	*P* < 0.001
Saturated fat	38.7 ± 8	28.8 ± 8.7	*P* < 0.001
Cholesterol	515.7 ± 215.3	399.3 ± 186.3	*P* < 0.001
Intake energy	2755.8 ± 10.7	2420 ± 392.3	*P* < 0.001
Calcium	724.8 ± 181	899.6 ± 414.9	*P* < 0.001
Mn	332.7 ± 49.6	335.7 ± 59	*P* = 0.48
Carbohydrate	332.4 ± 47.4	308.7 ± 48.1	*P* < 0.001
Protein	112 ± 22.9	109.4 ± 20.6	*P* = 0.12
Vitamin C	59.5 ± 27.2	76.8 ± 44.4	*P* < 0.001
Dietary fiber	9.8 ± 2.7	12 ± 4.6	*P* < 0.001
Intake sodium	3599.7 ± 258.4	2654.4 ± 540.6	*P* < 0.001
Knowledge	4.5 ± 11.3	3.2 ± 15.1	*P* < 0.001

**Table 3:** Correlation of systolic blood pressure with the variables of dietary behavior in controlled hypertension group.

	Systolic BP	Fat	Saturated fat	Cholesterol	Intake energy	Calcium	Mn	Carbohydrate	Vitamin C	Dietary fiber	Sodium
Systolic BP	1										
Fat	0.347^*∗*^	1									
Saturated fat	0.408^*∗*^	0.808^*∗*^	1								
Cholesterol	0.132^*∗*^	0.427^*∗*^	0.361^*∗*^	1							
Intake energy	0.288^*∗*^	0.827^*∗*^	0.694^*∗*^	0.335^*∗*^	1						
Calcium	−0.292^*∗*^	0.107^*∗*^	−0.214^*∗*^	0.09	0.172^*∗*^	1					
Mn	−0.160^*∗*^	−0.01	−0.04	−0.170^*∗*^	0.364^*∗*^	0.287^*∗*^	1				
Carbohydrate	0.115^*∗*^	0.249^*∗*^	−0.269^*∗*^	0.01	0.719^*∗*^	0.106^*∗*^	0.587^*∗*^	1			
Vitamin C	0.384^*∗*^	−0.295^*∗*^	−0.303^*∗*^	−0.142^*∗*^	−0.183^*∗*^	0.153^*∗*^	0.391^*∗*^	0.04	1		
Dietary fiber	−0.381^*∗*^	−0.306^*∗*^	−0.355^*∗*^	−0.185^*∗*^	−0.08	0.191^*∗*^	0.553^*∗*^	0.230^*∗*^	0.631^*∗*^	1	
Sodium	0.442^*∗*^	0.448^*∗*^	0.573^*∗*^	0.204^*∗*^	0.503^*∗*^	−0.258^*∗*^	−0.04	0.328^*∗*^	−0.475^*∗*^	−0.520^*∗*^	1

^*∗*^Correlation is significant at the 0.01 level (2-tailed).

**Table 4:** Correlation of systolic blood pressure with the variables of dietary behavior in uncontrolled hypertension group.

	Systolic BP	Fat	Saturated fat	Cholesterol	Intake energy	Vitamin C	Dietary fiber	Sodium
Systolic BP	1							
Fat	−0.002	1						
Saturated fat	−0.04	0.748^*∗*^	1					
Cholesterol	−0.04	0.066	0.123	1				
Intake energy	−0.04	0.661^*∗*^	0.567^*∗*^	0.046	1			
Vitamin C	−0.02	0.06	0.008	−0.07	0.257^*∗*^	1		
Dietary fiber	−0.006	0.121	0.031	0.006	0.438^*∗*^	0.534^*∗*^	1	
Sodium	0.847^*∗*^	0.06	0.019	−0.07	0.159^*∗*^	0.03	0.07	1

^*∗*^Correlation is significant at the 0.01 level (2-tailed).

**Table 5:** Multiple linear regressions of dietary variables in predicting systolic blood pressure in the patients of both groups.

	Model		Beta	*t*	*P* value	Adjusted *R* ^2^
Controlled group	1	Sodium	0.442	10.2	*P* < 0.001	19.5

Controlled group	2	Sodium	0.355	8.4	*P* < 0.001	23.4
		Nutritional awareness	−0.322	−7.6	*P* < 0.001	

Controlled group	3	Sodium	0.125	2.2	0.02	30.6
		Nutritional awareness	−0.263	−6	*P* < 0.001	
		Saturated	0.06	0.9	0.33	
		Vitamin C	−0.150	−3.2	*P* < 0.001	
		Calcium	−0.188	−4	*P* < 0.001	
		Intake energy	0.180	2.7	0.006	

Uncontrolled group	1	Sodium	0.847	24.4	*P* < 0.001	71.7

Uncontrolled group	2	Sodium	0.590	15.5	*P* < 0.001	80.4
		Nutritional awareness	−0.394	−10.4	*P* < 0.001	

Uncontrolled group	3	Sodium	0.621	16.9	*P* < 0.001	83
		Nutritional awareness	−0.368	−9.9	*P* < 0.001	
		Carbohydrate	−0.200	−6.1	*P* < 0.001	
		Dietary fiber	0.115	3.5	*P* < 0.001	
